# The AHR Signaling Attenuates Autoimmune Responses During the Development of Type 1 Diabetes

**DOI:** 10.3389/fimmu.2020.01510

**Published:** 2020-08-07

**Authors:** Tiantian Yue, Fei Sun, Chunliang Yang, Faxi Wang, Jiahui Luo, Ping Yang, Fei Xiong, Shu Zhang, Qilin Yu, Cong-Yi Wang

**Affiliations:** Center for Biomedical Research, NHC Key Laboratory of Respiratory Diseases, Tongji Hospital Research Building, Tongji Hospital, Tongji Medical College, Huazhong University of Science and Technology, Wuhan, China

**Keywords:** aryl hydrocarbon receptor, T1D, immune response, AHR Ligands, therapeutic target

## Abstract

The aryl hydrocarbon receptor (AHR) is a ligand-activated transcriptional factor widely expressed in immune cells. Its ligands range from xenobiotics and natural substances to metabolites, which renders it capable of sensing and responding to a variety of environmental cues. Although AHR signaling has long been recognized to be implicated in the pathogenesis of autoimmune disorders, such as rheumatoid arthritis (RA), colitis, and systemic lupus erythematosus (SLE), its effect on the pathogenesis of type 1 diabetes (T1D) remains less understood. In this review, we intend to summarize its potential implication in T1D pathogenesis and to sort out the related regulatory mechanisms in different types of immune cells. Emerging evidence supports that β cell destruction caused by autoimmune responses can be rectified by AHR signaling. Upon activation by its ligands, AHR not only modulates the development and functionality of immune cells, but also suppresses the expression of inflammatory cytokines, through which AHR attenuates autoimmune responses during the course of T1D development. Since AHR-initiated biological effects vary between different types of ligands, additional studies would be necessary to characterize or *de novo* synthesize effective and safe ligands aimed to replenish our arsenal in fighting autoimmune responses and β mass loss in a T1D setting.

## Introduction

Type 1 diabetes (T1D) is an autoimmune disorder stemming from the destruction of the pancreatic β cells by autoreactive immune cells, which leads to the absolute deficiency of circulating insulin. The pathogenesis underlying T1D is multifactorial, and involves a genetic predisposition coupled with environmental triggers (1, 2). According to the latest International Diabetes Federation (IDF) Diabetes Atlas, the number of young patients under 20-years-old living with T1D worldwide is more than 1.1 million, which is twice the number calculated in the previous atlas (3). Since the increased rate is much higher than the estimated genetic contribution, epigenetic factors, especially environmental triggers, are increasingly gaining more attention (4). For example, certain viral infections and/or improper medications could exacerbate T1D progression by damaging β cells (5, 6), while microorganisms may differentially affect T1D incidence and severity by modulating the intestinal environment and producing metabolic byproducts (7, 8).

The aryl hydrocarbon receptor (AHR) is a ligand-activated transcriptional factor capable of sensing and responding to a variety of environmental cues (9, 10), which could serve as a bridge to link environmental triggers to the pathogenesis of T1D. Indeed, compounds deriving from either exogenous milieu or emerging as endogenous metabolic byproducts could ligate to AHR, thereby eliciting cellular adaptive responses via different signal pathways (11). Particularly, AHR was initially recognized to be activated by the toxic xenobiotics associated with the detoxifying processes (12). However, subsequent studies further revealed that many additional harmless chemical substances could also effectively induce AHR activation to regulate immune responses (13). Moreover, accumulated evidence supports that AHR regulates not only innate immune cells, but also adaptive immune cells, by affecting intrinsic cell signaling events, cytokine secretion profiles, and inter-cellular communication processes.

Although AHR could be a pivotal environmental sensor to regulate immune responses, its role in T1D pathogenesis, however, is less appreciated. We thus in this review intend to summarize its potential involvement in T1D pathogenesis and to sort out the related regulatory mechanisms in different types of immune cells. We also highlight the feasibility of targeting AHR-related pathways to develop therapeutic strategies against T1D in clinical settings.

## AHR Structural Properties and Its Expression Patterns in Immune Cells

Aryl hydrocarbon receptor belongs to a family of basic helix-loop-helix/Per-Arnt-Sim (bHLH/PAS) transcription factors. The bHLH motif locates in the N-terminal of the AHR protein, containing a basic-region (b) and a helix-loop-helix region (HLH), which are involved in DNA-binding and protein-protein interaction, respectively. The PAS domain is a stretch of 200–350 amino acids, which allows AHR to have a high affinity to dimerize with its working partner, the aryl hydrocarbon receptor nuclear translocator (ARNT) (9, 14). Moreover, the C-terminal of the AHR protein contains a glutamine-rich (Q-rich) domain, which participates in co-activator recruitment and transactivation (15). As a ligand-dependent transcriptional factor, AHR has been recognized as being capable of modulating target gene expression. Once activated by its cognate ligands, AHR dissociates from its chaperones and translocates into the nucleus, where it forms a heterodimer with ARNT (AHR-ARNT) to transcribe the expression of target genes (16, 17).

In vertebrates, AHR is widely expressed in multiple cell types and is involved in the regulation of fundamental cellular processes, such as cell proliferation, differentiation, and stress responses. Immune cells are particularly enriched with AHR expression. For instance, AHR is highly expressed in Th17 cells and functions as a typical marker for its non-pathogenic population (nTh17) (18). Similarly, AHR expression is essential to support the survival of certain innate immune cells, such as TCRγδ T cells and innate lymphoid cells (ILCs) (19). Furthermore, AHR has been noted to be expressed in regulatory T cells (Tregs), dendritic cells (DCs), macrophages, and ILCs in intestinal mucosa and lamina propria (LP). These lines of evidence indicate that AHR is abundant in immune cells and acts as an important immune regulator.

## The Exogenous and Endogenous Ligands for AHR Activation

Aryl hydrocarbon receptor was originally recognized as a receptor for 2,3,7,8-tetrachlorodibenzo-p-dioxin (TCDD), a xenobiotic toxicant commonly found in industrial leakage, fossil fuel burning, medical waste, and traffic pollution (20, 21). TCDD possesses a high affinity to AHR, and therefore is able to alter the expression pattern of genes in embryonic stem cells (22). In this case, AHR serves as a characteristic receptor to sense small environmental molecules that harbor an aryl hydrocarbon structure (23). However, other than those exogenous xenobiotics, many endogenous and natural compounds are also identified to be potent ligands for AHR (13).

The typical endogenous candidate is a class of metabolic intermediates generated from tryptophan. Among them, kynurenine (Kyn), a tryptophan metabolite catalyzed by the indole 2,3-dioxygenase (IDO) and tryptophan 2,3-dioxygenase (TDO), has been the most studied (24). IDO1 is a rate-limiting enzyme for kynurenine production and is readily induced by inflammatory cytokines such as IFN-γ. Excessive IDO1 activity would exhaust endogenous tryptophan, thereby leading to the attenuated activation and proliferation of antigen-specific T lymphocytes, which serves as a negative feedback mechanism to prevent sustained immune responses (25, 26). Moreover, the amplified Kyn-AHR signaling induces tolerogenic dendritic cells (TolDCs) and regulatory T cells (Tregs), which further repress the ongoing inflammatory responses (27, 28). Therefore, altered IDO1-Kyn axis has been noted in a variety of disorders, such as tumors, neuronal abnormalities, and autoimmune diseases (24).

Diet-derived compounds constitute another category of AHR ligands. They come from fruits and plant-related products, and especially from cruciferous vegetables that are rich with glucosinolates. Following dietary uptake, glucosinolates can be degraded into indole-3-carbinol (I3C), which then undergoes condensation in the acidic stomach environment and drives the generation of 3,3′-diindolylmethane (DIM), indole [3,4-b] carbazole (ICZ), and [2-(indol-3-ylmethy)-indol-3-yl] indol-3-ylmethane (LTr1) (29). By activating AHR, I3C, together with its derivatives, facilitates a gut microenvironment for alerting the hosts to distinguish the nutrients constantly infested from those potentially harmful pathogens (30).

Some natural microbial metabolites can also bind to and activate AHR. For example, resident *Escherichia coli* utilizes tryptophan in the intestinal lumen to generate AHR ligands such as indole-3-propionic (IPA) and indole-3-aldehyde (IAld), which then promotes intestinal epithelial cells to secrete cytokines and initiate an anti-inflammatory response (31–33). In this case, AHR functions as a gut sensor to adjust the homeostasis of the gut microenvironment for protecting the host from intestinal inflammation.

Collectively, AHR is widely expressed in immune cells and exhibits ligand-dependent properties. The source of AHR ligands is diverse, including not only the xenobiotics but also natural substances and metabolites (Table 1). These discoveries further support that AHR acts as a bridge to closely connect environmental insults with homeostatic immune responses in daily life.

**TABLE 1:** The effect of AHR ligands on different cell types.

Sources	Ligands	Targeted cells	Function
Exogenous substances	TCDD	Treg	Treg generation
		ES cell	Inhibition of ES cell differentiation
	10-CI-BBQ	CD4^+^ Nrp1^+^ Foxp3^–^ RORγt^+^ cell	Attenuation of cell formation
	FICZ	Th17, Treg	Th17 generation; Foxp3 expression

Dietary	Glucosinolate	ILCs	Expansion of RORγt^+^ ILCs
	I3C	ILCs, IELs	Formation of lymphoid follicles, IL-22 production
	DIM	ILCs, IELs	Maintenance of IELs;
	ICZ	Th22, iNKTs	Epithelial cell proliferation
	LTr-1	γδT cells	Surveillance of microbial load;

Endogenous metabolites	Kyn	Treg, DC	Treg differentiation; Tolerogenic DC generation
	ITE	DC	Induction of tolerogenic DC
	KA	Cancer cell, Macrophage	IL-6 production

Microbiome	IPA	Intestinal epithelial cell	Activation of IL-10 signaling
	IAld	Astrocyte	Regulation of IFN-I signaling in astrocytes

*TCDD: 2,3,7,8-tetracholrodibenzo-p-dioxin; 10-CI-BBQ: 10-chloro-7H-benzimidazo [2,1-a] benzo [de] iso-quinolin-7-one; FICZ:6-formylindolo[3,2-b]carbazole; I3C: indole-3-carbinol; DIM: 3,3′-di-indoly-methane; ICZ: indole [3,4-b] carbazole; LTr-1: [2-(indol-3-ylmethy)-indol-3-yl] indol-3-ylmethane; Kyn: kynurenine; ITE: 2-(1′H-indole, 3′carbonyl) thiazole-4-carboxylic acid methyl ester; KA: Kynurenic acid; IPA: indole-3-propionic IAId: indole-3-aldehyde; ES: embryonic stem; ILCs: innate lymphoid cells; IELs: intraepithelial lymphocytes; DC: dendritic cells.*

## AHR Regulates Innate Immune Response During T1D Development

The initiation of an autoimmune response during the course of T1D development is complicated. It is believed that once the immune system loses tolerance to self β cells, the pathogenic process begins. This event, however, happens much earlier than the visualized clinical symptoms, and importantly, autoimmune responses and β cell dysfunction are tangled together to push T1D into an irreversible dead end.

**FIGURE 1:**
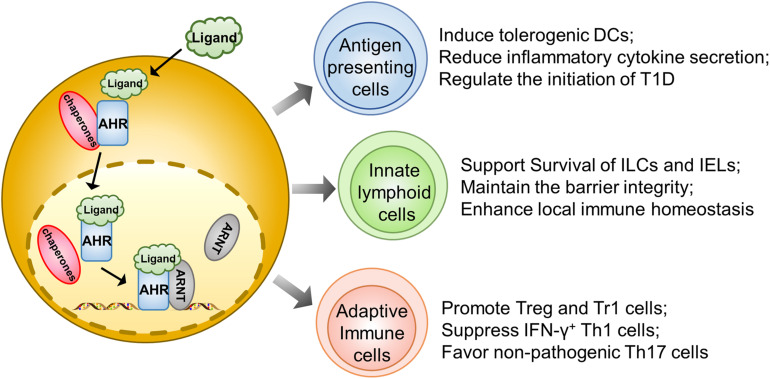
AHR signaling regulates autoimmune responses during the course of T1D development. Upon ligand-initiated activation, AHR dissociates from its chaperones and translocates into the nucleus, where it forms a heterodimer to transcribe genes essential for the development and functionality of different types of immune cells. Therefore, AHR signaling could exhibit a protective effect on the initiation and progression of T1D by acting on the antigen presenting cells (e.g., DCs and Macrophages), gut innate immune cells (ILCs, IELs, and γδT cells) and adaptive immune cells (regulatory T cells, Tr1 cells, Th1 cells, and Th17 cells).

Dendritic cells play an essential role in initiating autoimmune responses against pancreatic β cells. In fact, they are one of the earliest islet infiltrating leukocytes and are critical for the activation of lymphocytes in the early insulitis stage (34). However, AHR was noted as being able to affect their function for antigen presentation and induction of T cell activation. For example, AHR activated by the diet-derived non-toxic endogenous AHR ligand, 2-(1′H-indole, 3′carbonyl) thiazole-4-carboxylic acid methyl ester (ITE), is associated with the induction of tolerogenic DCs, which exhibits a phenotype of decreased CD86 expression, increased CD103 expression, and diminished secretion of inflammatory cytokines, coupled with the capability to induce the generation of Foxp3^+^ Tregs (28). Therefore, those non-toxic endogenous AHR ligands could have the potential to prevent and treat autoimmune disorders (Figure 1).

Similar to DCs, macrophages also play a predominant role in T1D initiation and progression by means of antigen presentation or production of inflammatory cytokines to destroy β cells (35). Generally, upon activation, AHR reduces IL-6 expression in macrophages to suppress immoderate inflammatory responses (36). Macrophages also manifest their endocytic function to timely remove the apoptotic cell debris. In T1D susceptible subjects, the accumulation of apoptotic β cells could serve as a significant source of autoantigens and damage-associated molecular patterns (DAMPs) (37). Indeed, macrophages isolated from NOD mice in a human T1D model could not efficiently remove apoptotic β cells during the course of β mass turnover, which caused apoptotic β cells to undergo a secondary necrosis associated with autoimmune initiation (38). Therefore, blockade of AHR signaling abrogates apoptotic cell induced IL-10 expression in macrophages coupled with a shift of tolerance toward a pro-inflammatory state (39) (Figure 1).

Innate lymphoid cells, originating from common lymphoid progenitors (CLPs), are recently discovered innate complements of T lymphocytes. Based on the difference in transcriptional factor, biological function, and cytokine profiling, ILCs are generally divided into ILC1, ILC2, ILC3, natural killer cells (NK), and LTi (40). ILCs diffusely exist in intestinal mucosa and LP, where they produce cytokines, such as IL-17A and IL-22, to maintain the barrier function and protect from pathogenic infection (41). Recent studies highlight the interrelationship between gut barrier integrity and autoimmune T1D. There is evidence that the pancreatic endocrine cells could cross-talk with gut microbiota through secretion of antimicrobial peptides (AMPs) induced by microbiota metabolites in NOD mice, which exerts an immunoregulatory function to halt the ongoing pancreatic inflammation (42). Indeed, impaired intestinal barrier would exacerbate the T1D process. For instance, colonic infection of young NOD mice by a bacterial pathogen disrupts the intestinal epithelial barrier and promotes invasive insulitis (43). Notably, AHR is found to be highly expressed in intestinal ILCs and necessary for the gut microenvironment remodeling. The population of ILCs specializes in the production of large amounts of IL22 and has often been called ILC22. Specifically, AHR could drive the development of ILC22 cells and the formation of postnatally developed cryptopatches and isolated lymphoid follicles (but not embryonically formed Peyer’s patches) *via* the induction of Notch, by which it promotes IL-22 secretion and surveillance of extracellular pathogens to support mucosal epithelial cell survival, proliferation, and anti-microbial function (41, 44). AHR could also maintain the viability of intraepithelial lymphocytes (IELs) as evidenced by the excessive microbial loads and vulnerability of epithelial cells following AHR deficiency, thereby leading to the over-activation of the local immune system (45). Therefore, the AHR-ILCs axis is essential to maintain gut integrity, which may impact T1D development *via* a crosstalk between the gut and pancreas (Figure 1).

## AHR Suppresses Effector T Cell Function During T1D Development

T cells are the main effectors responsible for β cell destruction, especially in the advanced stage. However, the T1D-prone NOD mice show reduced activity of AHR (low affinity AHR^*d*^ genotype) as compared to that of B6 mice (high affinity AHR^*b*^ genotype). TCDD, which increases Foxp3^+^ T cell proportion *via* activating AHR, could prevent diabetes development in NOD mice (46). Similarly, the exogenous AHR affinitive ligand, 10-chloro-7H-benzimidazo [2,1-a]benzo[de]isoquinolin-7-one (10-CI-BBQ), exhibits protection against T1D in NOD mice by inhibiting the formation of disease-associated CD4^+^ Nrp1^+^ Foxp3^–^ RORγt^+^ cells. Notably, such an effect relies on AHR activation but is independent of Foxp3^+^ regulatory T cells (47). Th1-related cytokines, such as IFN-γ and TNF-α, are thought to be the major effective molecules leading to β cell death (48), while T cells deficient in *AHR* produce more IFN-α, IFN-γ, and IL-12 (49). Although the exact role of Th17 in T1D development is under debate, emerging evidence suggests that the IL-23-induced pathogenic Th17 cells contribute more to T1D pathogenesis than the conventional Th17 cells, and AHR serves as a characteristic marker of the latter (50, 51). In human T cells, it was also found that AHR activation in the presence of TGF-β induces functional Treg cells to suppress pathogenic responder T cells, which is consistent with the results from mouse studies (52). Altogether, AHR could suppress effector T cell function through directly acting on effector T cell subsets or indirectly inducing Treg cells (Figure 1).

## The Mechanisms Underlying AHR Regulation of Immune Cells

As a transcriptional factor, AHR is efficient to modulate the expression of lineage specific transcriptional factors in immune cells. For example, AHR favors Treg induction and development by directly binding to the *Foxp3* promoter upon TCDD-induced activation (53). AHR is also highly expressed in Th17 cells and is essential for its differentiation and effector function. Specifically, RORγt, a downstream target of AHR, functions as a critical transcriptional factor for IL-17 producing cells (54). AHR can also form a complex with RORγt to enhance the transcription of IL-22, which is of great importance for the function of many innate immune cells including ILC3s, γδT cells, and LTi-like cells (55). These types of RORγt^+^ cells are abundantly located in the mucosal linings of the body (e.g., intestine and lung) to maintain the defensive barrier function via secreting IL-22. In the absence of AHR, RORγt^+^ ILCs are susceptible to apoptosis and they produce a lesser amount of IL-22 (55, 56). Other than RORγt, the synergistic effect is also observed in type 1 regulatory T cells (Tr1 cells), which are Foxp3 negative and produce high levels of interleukin 10 (IL-10), and are decreased in adult-onset T1D patients (57–59). C-Maf is a transcription factor encoded by the *Maf* (musculoaponeurotic fibrosarcoma) gene. In addition to serving as an oncogene, c-Maf has noted to be involved in cell differentiation processes. Specifically, c-Maf transactivates the expression of IL-10 and IL-21, which are crucial for Tr1 induction (60). IL-27, an essential cytokine for Tr1 development, possesses the ability to activate AHR, which then couples with c-Maf to promote IL-10 expression, thereby ameliorating autoimmune responses in an EAE setting (61).

The signal transducer and activator of transcription (STAT) family consists of seven members (STAT1-4, STAT5a, STAT5b, and STAT6) that are indispensable for the functionality of immune cells. Mutations with gain of function in human STAT1 and STAT3 are related to T1D susceptibility by perturbing the equilibrium between Th1, Th17, and regulatory T cells (62). AHR has also been found to repress STAT1 phosphorylation by forming an AHR-STAT1 complex, thereby reducing the transcription of IFN-γ (49). Specifically, 6-formylindolo[3,2-b]carbazole (FICZ) facilitates naïve T cell differentiation toward Th17 cell lineage, during which activated AHR interacts with STAT1 to restrain its activity, leading to the stabilization of Th17 identity while repressing Th1 program (63). Similarly, LPS stimulation enhances AHR expression in macrophages, which in turn forms a complex with either nuclear factor-kappa B (NF-κB) or STAT1 to inhibit IL-6 transcription, by which AHR signaling alleviates inflammatory responses (36). Upon LPS-stimulated activation, AHR also promotes IL-10 transcription by activating the Src-STAT3 pathway to attenuate the secretion of other inflammatory cytokines in macrophages (64).

**FIGURE 2:**
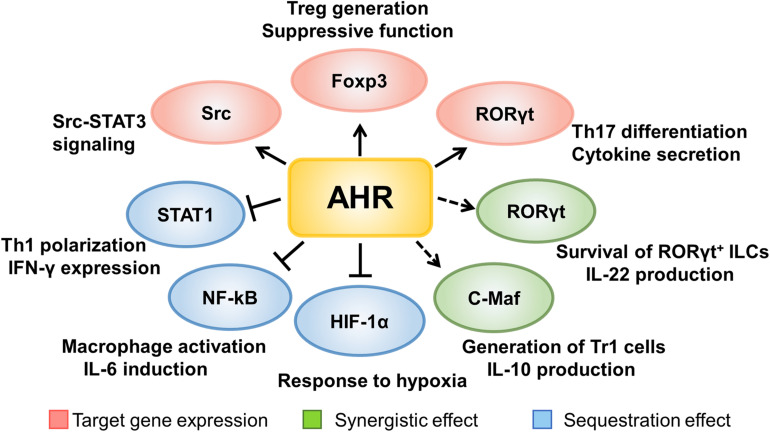
The mechanisms underlying AHR signaling regulation of immune cells. AHR signaling regulates the development and functionality of immune cells either by directly binding to the downstream target genes, or by forming a complex with other transcriptional factors to control the expression of critical genes necessary for autoimmune responses during the course of T1D development.

Taken together, AHR signaling significantly regulates the development and functionality of immune cells either by directly binding to the downstream target genes, or by forming a complex with other transcriptional factors to control the expression of critical genes necessary for autoimmune responses during the course of T1D development (Figure 2).

## Conclusive Remarks and Perspectives

Although AHR signaling has long been recognized to be implicated in the pathogenesis of autoimmune disorders, such as rheumatoid arthritis (RA), colitis, and systemic lupus erythematosus (SLE) (39, 65, 66), its effect on T1D pathogenesis remains less understood. Given the fact that AHR is widely expressed in innate immune cells (e.g., DCs, macrophages, ILCs, IELs, and iNKTs), effector T cells (e.g., Th1 and Th17 cells), and anti-inflammatory cells (e.g., tolerogenic DCs, Tr1, and Tregs), we thus in the present review discussed its potential role in T1D pathogenesis. In general, T1D development involves β cell destruction resulting from excessive activation of autoreactive immune cells along with repressed activity of regulatory cells. Emerging evidence supports that β cell destruction caused by this disrupted immune homeostasis can be finely rectified by AHR signaling. Upon activation by its ligands, AHR not only modulates the development and functionality of immune cells, but also suppresses the expression of inflammatory cytokines, through which AHR attenuates autoimmune responses during the course of T1D development. In line with the observation, IDO1, an important enzyme for endogenous AHR ligand production, is also implicated in T1D pathogenesis. For example, a chimeric protein constituted by proinsulin and cholera toxin B subunit (CTB-INS) is able to promote IDO1 expression, which then enhances tryptophan degradation to modulate tolerogenic properties in DCs, thereby halting T1D progression in NOD mice (67).

It is worthy of note that we did not discuss the possible implication of AHR signaling in β cell viability and functionality. However, there is feasible evidence that *Arnt* (also called HIF-1β) forms a heterodimer with AHR to affect β cell homeostasis. Specifically, β cells deficient in *Arnt* manifest impaired glucose stimulated Ca^2+^ signaling and insulin secretion (GSIS), which has been confirmed by studies in human islets using an *Arnt* siRNA (68). Similarly, *Arnt-/-* islet grafts exhibit increased β cell apoptosis along with a reduced β mass following transplantation (69). Furthermore, HIF-1α, another partner of *Arnt*, is up-regulated in infiltrated islets, which then transcribes the expression of anti-apoptotic genes in β cells to prevent T1D progression in NOD mice (70–72). However, it is quite possible that AHR would compete with HIF-1α to form a heterodimer with *Arnt*, and therefore, the exact regulatory mechanism underlying AHR signaling in β cells demands further studies.

In general, AHR acts as a molecular sensor by employing various exogenous and endogenous ligands to respond to the external and/or internal signals. These unique properties render it a druggable target to rectify altered immune homeostasis in the setting of autoimmune disorders. Indeed, mice supplemented with I3C, an AHR ligand, are protected from DSS-induced colitis (45). Since phytochemicals such as polyphenols and glucosinolates are enriched in grains, and cruciferous vegetables are abundant with I3C, a diet formulated with those components could be a good approach to prevent pathogen invasion and enhance immune homeostasis (55). Similarly, the indole derivatives could be applied to improve insulin sensitivity and alleviate chronic inflammation through AHR signaling (73).

In summary, accumulated evidence supports that AHR signaling negatively regulates the initiation and progression of autoimmune responses in T1D setting. AHR possesses a variety of exogenous and endogenous ligands, and the resulting biological effect varies depending on the ligand employed. These unique properties rendered it a viable therapeutic target. However, additional studies are needed to characterize or *de novo* synthesize effective and safe ligands aimed to replenish our arsenal in fighting autoimmune responses and β mass loss in a T1D setting.

## Author Contributions

TY and FS wrote the manuscript and prepared the figures. CY, FW, and JL collected and analyzed the information. PY, FX, and SZ reviewed the manuscript. C-YW and QY supervised the conception and writing of the manuscript. All authors contributed to the article and approved the submitted version.

## Conflict of Interest

The authors declare that the research was conducted in the absence of any commercial or financial relationships that could be construed as a potential conflict of interest.
